# Supplementary material for: Population Genetic Divergence and Environment Influence the Gut Microbiome in Oregon Threespine Stickleback
Source: Genes (Basel). 2019 Jun 26;10(7):484. doi: 10.3390/genes10070484 (PMC6678413; doi:10.3390/genes10070484)
Supplement: Supplementary file 1 [file genes-10-00484-s001.pdf]

LIST OF SUPPLEMENTAL FIGURES

Supplemental Figures Page

1. PCoA ordination of a Bray-Curtis dissimilarity matrix using square root and relative abundance transformed ASV counts.

2. Gut microbiome composition among threespine stickleback across Oregon for different dissimilarity metrics.

3. Proportion of variation explained by PCoA axes.

4. Bar plot of relative abundances of gut bacteria community at phylum level.

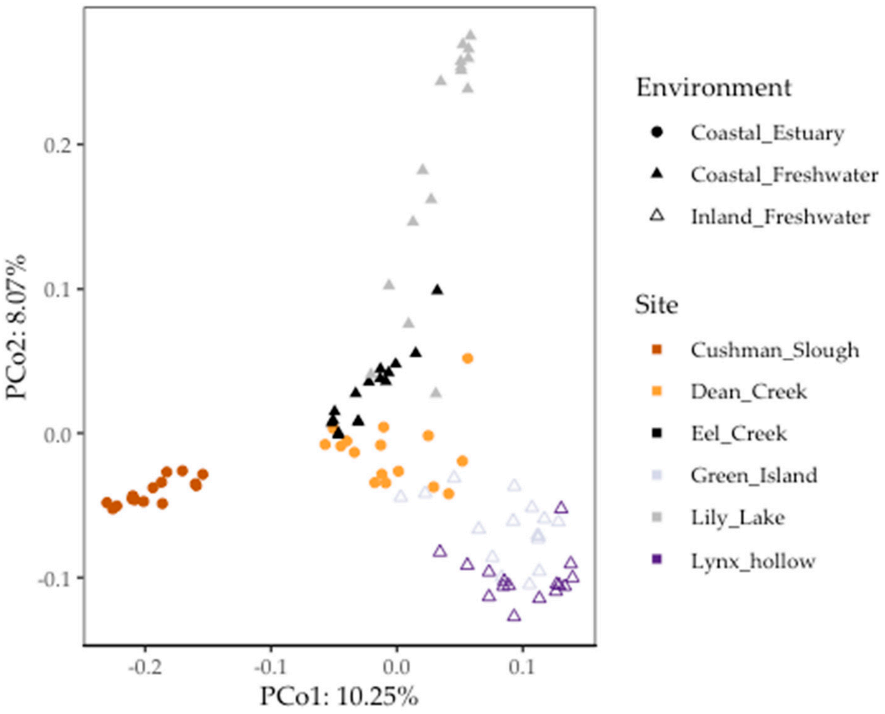

Supplementary Figure S1. PCoA ordination of a Bray-Curtis dissimilarity matrix using square root and relative abundance transformed ASV counts. PCoA axes 1-2 are plotted here. Each point represents a fish gut microbial community. Shape fill represents whether fish were in the Willamette Basin ("Inland") or in watershed along the coast ("Coastal"). Colors represent collection sites ("populations") in Oregon.

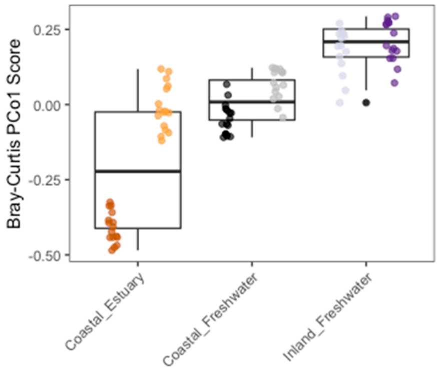

(**a**)

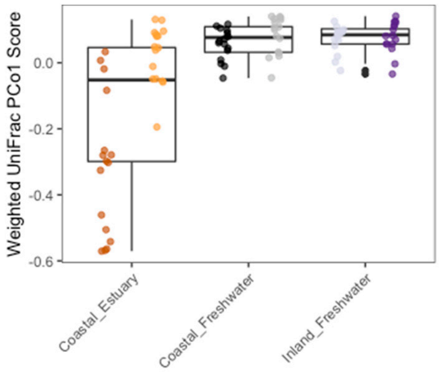

(**b**)

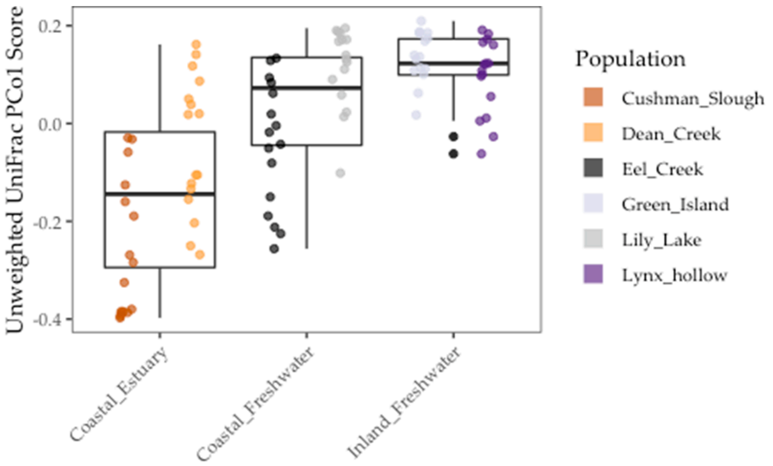

(**c**)

Supplementary Figure S2. Gut microbiome composition among threespine stickleback in Oregon varied in terms of PCoA of: (**a**) Bray-Curtis dissimilarity; (**b**) Weighted UniFrac distance; (**c**) Unweighted UniFrac distance. Only PCoA axis 1 is shown here for all three approaches. Bray-Curtis dissimilarity was square root and relative abundance transformed. Colors represent collection sites ("populations"). Mid-box lines are pooled means of major environment (e.g. Coastal Freshwater"). Box whiskers are pooled standard deviation of major environments as well.

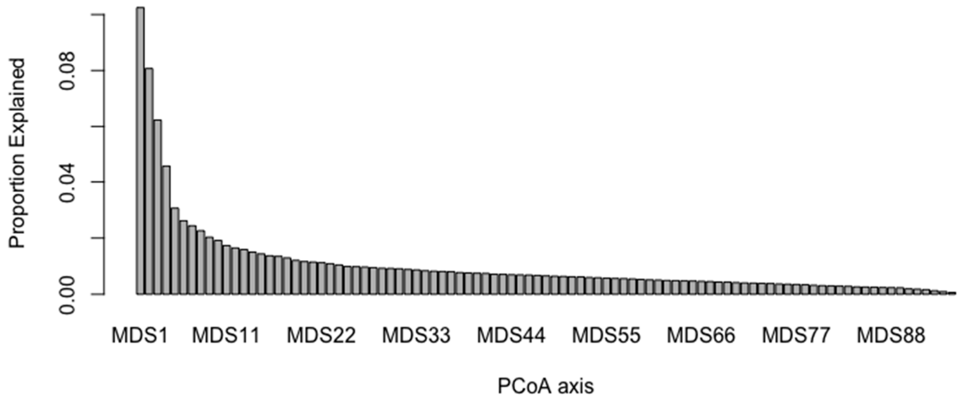

Supplementary Figure 3. Each PCoA axis explained only a small proportion of the overall variation in gut communities among fish.

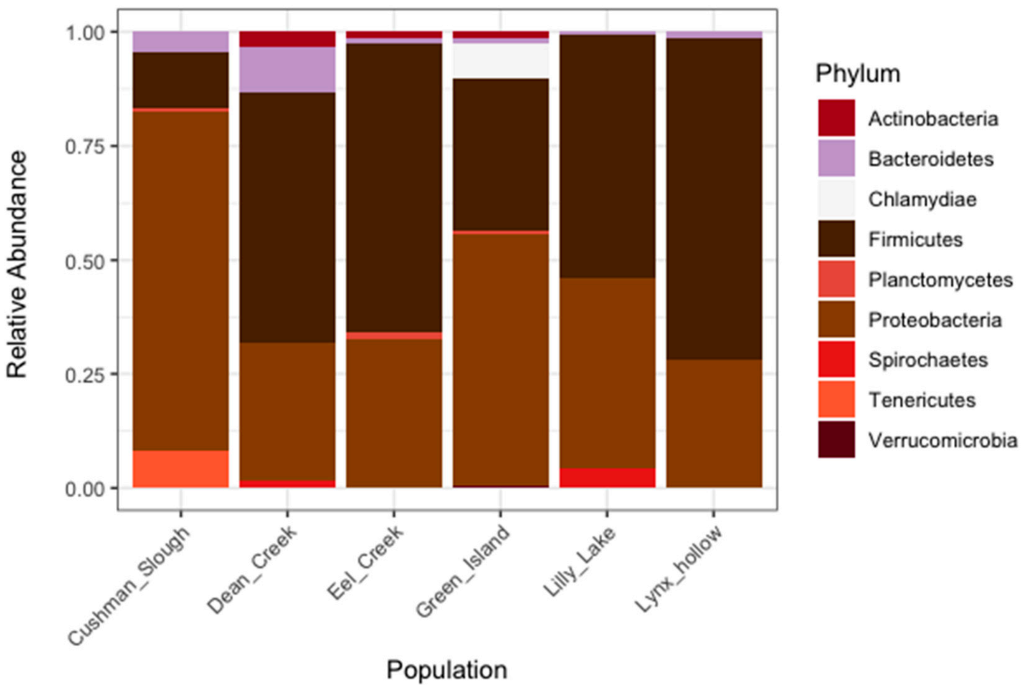

Supplementary Figure 4. Phyla that were found with a relative abundance > 10% in the gut microbiome in threespine stickleback population in Oregon dominated by 10 phyla. Colors represent different phylum. Bars are collection sites ("populations").
